# The New York Institute of Stomatology

**Published:** 1902-03

**Authors:** 


### Reports of Society Meetings.

### THE NEW YORK INSTITUTE OF STOMATOLOGY.

A REGULAR meeting of the Institute was held on Friday evening, November 8, 1901, at the office of Dr. C. B. Parker, 167 Remsen Street, Brooklyn, N. Y., the President, Dr. J. Morgan Howe, in the chair.

The minutes of the previous meeting were read and approved.

Owing to the length of the programme, the communications on theory and practice were omitted.

Dr. Charles F. Allan, of Newburgh, N. Y., read a paper entitled "What should be the Relation of our Government to the Dental Profession?"

(For Dr. Allan's paper, see page 149.)

*The President.*—Gentlemen, you have this very interesting paper of Dr. Allan's before you for discussion. I trust you will discuss the subject thoroughly and try to make your suggestions practical. We have the honor of having with us Drs. Jamison and Bristowe. We shall be very glad to hear from them.

### DISCUSSION.

*Dr. A. T. Bristowe.*—This whole question opens up a very broad subject, the subject of protection, in which I have long been interested. As long as this is the policy of the government, how can we as physicians and dentists expect the government to make us a favored class and exempt us from the general policy of protection to which all arts and sciences pay tribute? So far as surgeons are concerned, there are very few things of which protection deprives us. About the only surgical instruments are the cystoscopes, and for the physicians microscopes and some few pieces of diagnostic apparatus.

Microscopes made on this side are not quite so satisfactory as those of foreign make, and all the better grades of oil-immersion lenses are made on the other side. It is no doubt a disadvantage and an injury to us to have to pay duty on these things, but at present I fail to see how any practical suggestion can be made whereby we can escape the payment of this duty. When we think of the trusts that protection has nourished, I confess the question is a difficult one. Take the steel trust. Steel is being sold across the water cheaper than in this country on account of protection. Do you suppose the steel trust is going to let anybody deprive it of its special advantage and influence? Not so long as there is any money to influence legislation. Take the pharmacists' trust. They represent a large amount of capital, and they will never voluntarily permit any change in the tariff which will affect them. It is a matter of fact that in the Committee of Ways and Means at Washington it is largely a scramble to see who shall get the biggest protection. It is purely a question of legislation, and that is a question of money and influence.

How are you going to attack the tariff? You will find that if any change is attempted, even in so small a matter as dental instruments, those to whom protection gives special privileges will fight any change as long as they can. If I were asked to make any practical suggestion in the matter, the only one I could offer would be "patience." I say this because some of the wisest statesmen are beginning to see that we cannot always live behind this Chinese wall of exclusion and yet sell our products to other nations while denying them our markets. Our late President McKinley referred to this in his last speech at Buffalo. Reciprocity is a new word in politics, but it may mark the beginning of a new era in trade methods. As our laws become more liberal, we as scientific men may expect more liberal treatment from the government, but not, I fear, until the necessity of a change in our present policy becomes evident to our legislators.

*Dr. S. E. Davenport.*—It seems to me that we are under obligation to Dr. Allan for pursuing this subject as thoroughly as he has, and giving us the result of his research and thought. The gentleman who last spoke is certainly most convincing, and the position he has taken I believe to be true. Probably this unjust taxation of chemicals and supplies is only a part of a general plan, and we can hardly expect any great change in this particular direction except through a change in the policy of the administration. There is hope for us and all like sufferers in the fact that the revenue of the government is largely in excess of its necessary expenditures, and the constant reader of the news of the day finds many suggestions from prominent men all through the country regarding the advisability of reducing the duties on a great many articles. I am very sure that Congress might be induced to make a considerable difference in favor of the things used by members of the great healing art. There are, in my mind, a few possibilities in this matter which may be worth following out. Certainly this paper ought to have a wide publication, and we should use our influence to bring the matter to the attention of those gentlemen who have influence with the Committee on Ways and Means at Washington. I shall hope that the Board of Directors of this society will take such action in this matter that other societies of dentists and if possible the larger medical societies will be enlisted in this movement.

*The President.*—It has been truly said that we suffer in common with others, and the tariff tax on our goods is only a sample of similar taxes on instruments of investigation, such as the microscope, and all kinds of instruments and books. We are under much obligation to Dr. Allan for having put the matter in such a clear and forcible shape, and I think the dissemination of these facts will do good in fostering a sentiment that will not continue to acquiesce in the policy that puts a tax on scientific progress, the advancement of knowledge, and the progress of the art of relieving suffering and the curing of disease.

*Dr. C. F. Allan.*—I was very much impressed with the remarks of the first speaker: they were certainly reasonable, and the argument was of great strength. My hope, as I thought I put it in my paper, lies along two lines: one being that scientific men, particularly those of the medical profession, are on a different plane and stand in a different light before the public than manufacturers, and should not be included with them in any argument.

I can readily understand the force of the position taken,—*i.e.*, that the manufacturers would hardly submit to any break in the line of protection, fearing that, the break once made, their interests would be the more likely to be put in jeopardy; but, assuming, as we have the right to assume, that as members of the healing art we should be considered as a class by ourselves, with separate and paramount interests, as I tried to make clear in my paper, I can but feel that much could be accomplished if the matter were presented before the proper committees of Congress in a clear manner and with the might of the whole medical fraternity behind it. In the line of dentistry my other hope comes from the fact that the tariff on dental goods does not protect and does not prohibit. It neither helps the manufacturer nor gives revenue to the government, and being so easily assailed is a great support to our stronger position that we should be relieved from the onerous tax because of its inherent injustice.

*Dr. Charles O. Kimball.*—I, too, wish to thank our essayist for the presentation of these facts to us. It seems to me that in view of any wrong of this kind,—and I do not wish to be considered as speaking as a free-trader or against the tariff as a whole—"that is another story"—since we represent the healing art in one of its departments, and as, under other governments, and even under our own government, such men have received some consideration,—this seems to be wrong. The only chance for our obtaining relief is along two lines. First, the line of information. The publication of this paper and this list of duties will set men to thinking who have not thought before except as a matter of course, so that perhaps we may be able to achieve some change in time. I think it will be a work, no doubt, of patience and long waiting. The other line is that of agitation. These two lines are the lines for the removal and obliteration of any wrong,—information and quiet, steady, and persistent agitation; and for that reason, Mr. President, we have to thank Dr. Allan for bringing this subject so clearly before us this evening.

On motion of Dr. George S. Allan, it was ordered that the printed tables of tariff rates of the different countries be mailed to the editors of the different dental journals of the United States and Canada.

*The President.*—We will now pass to a consideration of Dr. Garrett Newkirk's paper on "Preventive Dentistry." We hope that all here present have read the paper to which attention was called in the notices. It is now before you for discussion.

(For Dr. Newkirk's paper, see *Dental Digest,* pages 743-749.)

DISCUSSION.

*Dr. S. E. Davenport.*—I have received the following communication from Dr. Lord:

I am sorry indeed that I cannot hear the discussions on the paper, the subject being one of the most important that ever comes before our society. I consider that the subject is well treated by the author of the paper, and it is to be hoped that very much more interest will be taken in it and attention paid to it in the near future than has been in the past.

Dr. Newkirk has expressed himself very simply and yet clearly. His theories are for the most part quite correct in my judgment, and his points well taken and practicable, and he has covered the ground so thoroughly that I feel that I can add but little that will be of interest or edifying.

Of course, as I understand the matter, preventive treatment of the teeth is almost entirely mechanical, and, as is suggested, in order to be successful should and must commence at a very early age, as soon as the deciduous teeth are erupted, and it is also suggested, and there can be no question as to the correctness of the view, that cleanliness is the real true foundation or remedy for prevention. It would seem to be very easy to apply it, but it is not done, and the question arises, Are not the dentists more at fault than their patients?

It is most probable that we have neglected our part of the work. The people, young and old, parents and those who have the care of young children, all must be taught how to clean the teeth and how to keep them clean, the surfaces that are in contact even more than the surfaces exposed to the natural friction occasioned by the use of the teeth. The reason is obvious. All this must be explained from time to time when it is observed that there is more or less neglect or misunderstanding. Then the means to be used must be furnished, and the patients instructed how to use them. We hear patients say that they do not understand how their teeth can decay, as they brush them so thoroughly. I have thought sometimes that if tooth-brushes had never been manufactured, other and more effective means would be resorted to for keeping the teeth clean and so preventing decay. I am a firm believer in the toothpick, particularly for the benefit of the gums. It cannot be passed between the teeth,—that is, between the whole length of the teeth,—but it can be passed between the teeth at the gum point, and so prevent the gum from becoming swollen and filling up the natural space. We often have patients come to us with the point of the gum between the teeth so congested and swollen that it is quite impossible to keep the teeth clean or use the floss-silk without much distress. If we find this condition of things, the first thing to be done is with a suitable instrument to remove the point of gum, and then we can show the natural space and speak of the importance of keeping that space free.

A suitably shaped wooden toothpick is probably one of the best, if not the best, means to use to keep the natural space between the teeth at the gum, and this will greatly aid in keeping the teeth clean at that point.

I have found great usefulness in passing an instrument between all the teeth where it is possible, and with a suitable instrument this can be done in nearly every case. The instrument should be nearly as wide as the length of the short teeth, in order to give proper strength, and the temper of the steel must be just suitable, or it will be a failure. This little operation is often very useful to the gums where there is congestion and to enable the silk to be passed more easily.

It is self-evident that the proper preventive means and appliances cannot and will not be used to advantage unless there should be a proper knowledge of what the result will be, and great interest felt in the result, all of which does not yet prevail, at least to any extent.

It is a good suggestion in the paper that the relation of the physician and the dentist should be such that they would act in concert in the use of preventive means, and this would awaken, or be likely to, a desire on the part of physicians to know more about the teeth than they now do, in many respects. There can be no question but that the dentist should be well informed in regard to diseases generally and their treatment.

*Dr. E. H. Babcock.*—I have read the paper through, and am very much interested in it. It seems to me that at the present time dentistry has accomplished about all it can by means of fillings. In order to preserve teeth we must resort to other means. There have been various ideas presented within the last few years. Dr. Black presented the idea of the extension of fillings which to my mind is chiefly correct. His idea is to bring the margin of the filling to a point where the food cannot collect or where it can be easily removed with the brush. Dr. Newkirk claims that a clean tooth never decays. If the teeth are thoroughly cleansed, not only by the patient but by the operator, at frequent intervals, their condition will be much improved. I do not quite agree with Dr. Black as to the extent to which he would carry his preparation of the enamel margins. I had a little girl brought into my office early this week. After examining her teeth I turned to the mother and said, "I believe you need a good scolding." The child was eight years old, and her teeth had been badly neglected. The mother said that she did not know a child needed to be brought to a dentist before the eighth year. I cleaned up the teeth and called attention to the sixth-year molars, which were so badly broken down that they needed to be extracted. To cite another case: about three years ago, just as I was leaving my office, a gentleman called with such a bad condition of the gums that they bled at the slightest touch. After working over him for two hours and removing a large amount of salivary calculus, I got the gums in a healthy condition.

Regarding the relations of the dentist to the general practitioner, my experience has been that the physician is very apt to look into the condition of the teeth of his patients, understanding that without sound teeth for mastication there cannot be good digestion.

*Dr. E. A. Bogue.*—Before speaking on the subject I would like to remind the last speaker that he is uttering a heresy when he talks of extracting the sixth-year molars in a little girl eleven or twelve years of age. He evidently is not aware that it is impossible to properly masticate the food after these teeth have been lost. I trust he will revise his remarks, leaving out this heretical doctrine.

I was greatly pleased to see Dr. Newkirk's paper. He seems to have carefully considered the views of Drs. Black, Williams, and Hopkins, gentlemen who have all taken a broader view than the field of practice which lies before them. It has been said, and perhaps truly, that reparative dentistry has gone as far as it can, as such, in the preservation of the teeth, but it must go farther than merely preserving the teeth that are brought to the dental specialist, and the general practitioner is going to aid us enormously in that work. I do not think we have learned more than the first faint glimmering of the truth as yet. One writer has gone so far as to claim that two persons contemplating marriage should be properly mated so far as the teeth are concerned. We as dentists seldom see a set of thoroughly good teeth. I have occasion to know that a great many of our profession are not aware that there is such a thing as a set of teeth so perfect that they will last through three score years and ten without the dentist's care. Yet such teeth exist. I have had occasion several times to allude to one gentleman that I have met who was fifty-two years of age, who never had had a brush in his mouth, and yet his mouth was perfectly clean and void of tartar. I have had patients come into my hands whose deciduous teeth were riddled with holes before they came through the gums, and one whose permanent teeth had from five to ten cavities in each of them. Now, at twenty-four years of age, she has never lost a tooth and has never had a toothache. But this is all mechanical repair. What can be done with such a child, between the hour of birth and the twelfth year of age, to get the enamel-organs in such a condition that they will perform their function properly? It seems to me that in one way our duty is clear,—that we should teach the mother of every child that comes into our hands how to keep that child's teeth clean so as to prevent decay, and if possible see that the physician keeps the child in such a condition that the fluids of the mouth shall not be productive of decay. It has been claimed by Dr. Michaels that the saliva is often in such a condition as to favor decay, and I think, not infrequently, we see children from eight to sixteen whose mouths are never in a healthy condition, because of improper food, improper mastication, and a failure to remove the débris of food, which, remaining, harbors the bacteria of decay. I do not know, Mr. President, if it is proper to make these rather crude remarks, as they have no especial bearing on Dr. Newkirk's paper.

*Dr. Babcock.*—I think Dr. Bogue would not have taken me to task regarding the suggested extraction of the sixth-year molars in the little girl of eight if he could have seen their condition. They were so extremely decayed that it seemed impossible to save them, and my idea was that the twelfth-year molars would come forward and take their place.

*Dr. Davenport.*—Dr. A. H. Brockway, our vice-president, has for years called attention to the fact that many valuable papers are read before dental societies and dismissed with but little discussion, either from lack of time or because the hearers were not prepared to discuss them, through failure, perhaps, of the Executive Committee to distribute digests of the paper beforehand.

Dr. Brockway has often suggested that our Executive Committee would do well for the Institute by selecting for discussion before this body valuable papers read before other societies, instead of giving their thought and effort to the securing of entirely new matter.

The paper entitled "Preventive Dentistry," read before the Illinois Dental Society last May by our member, Dr. Garrett Newkirk, of Los Angeles, Cal., and published in the October *Dental Digest,* seems to me to belong to this class of papers, much too valuable to be allowed to pass into dental literature without discussion.

Dr. Newkirk has shown great ability in bringing together in a concise way many practical thoughts, which if followed out in practice would result in service of the highest class to the needs of patients and, better even than that, in a prevention of a considerable proportion of their usual needs.

This whole subject of prevention, in the various lines in which it is being thought out seems to me to be *the* important subject of the time, and it behooves us all to give such attention to it as will enable us to derive all possible benefit from it, thereby doing our duty to ourselves and our patients.

It seems to me that the discussion of Dr. Newkirk's paper should be approached from two directions: first, the consideration of such plans and methods for the prevention of decay as may be taught by prophylaxis, hygiene, etc.; and second, such methods as will anticipate and make improbable more serious trouble.

No doubt we all agree with Dr. Newkirk that a clean tooth-surface will not decay, and, following that thought, our duty is not only to instruct all patients how to do the best possible cleansing for themselves, but also to see them frequently and make use of such prophylactic measures as shall prevent the retention of tartar and food between the teeth. Probably no system is as valuable for this purpose as that so ably and enthusiastically advocated by Dr. D. D. Smith, of Philadelphia. The condition of the teeth of the patients Dr. Smith so kindly exhibited to us last June was certainly a revelation, and probably no cleaner mouth was ever presented for examination before a dental society.

Whether it is necessary or desirable for patients to present themselves as frequently as Dr. Smith's are required to, every operator must decide for himself, but the value of the method is beyond all question, and no practitioner who would do his best for his patients will dispense with its use.

Some dentists have made use of the method for preventing decay advocated by the late Dr. Hart, of San Francisco, with considerable satisfaction, erosion particularly being prevented or retarded by the thorough application of formalin, dioxygen, etc. Whatever aid we can get from these various methods of prevention is just so much gain for our patients and for ourselves.

The late Dr. Stebbins, of Shelburne Falls, Mass., was certainly a benefactor of the human race, and made for himself a very high place among dentists by reason of his suggestion and of his careful experiments with the use of silver nitrate to prevent the extension of decay in the small cavities of children's teeth, as well as on the softening surfaces of the back teeth of adults, all of which it does effectually; and were it not for the discoloration caused by its application, we would derive great benefit from its use upon sensitive and eroding teeth in the front of the mouth.

One of the recognized needs of the time is a chemical, or the combination of two or more, which will give the good results of silver nitrate without its discoloration.

Leaving now the consideration of the various methods of preventing the beginning of decay, I will pass on. I would like to say just a word on the methods which should be used to retard decay and to prevent more serious trouble when that decay exists. It gives me pleasure to call attention to the fact that Dr. E. A. Bogue has, since I have known him, earnestly advocated the filling of the smallest cavities in the permanent teeth of children. Of course, there are small cavities in the teeth of adults that it is quite proper to leave unfilled, but with the teeth of children, with their different environment, it is certainly to the advantage of these patients that the slightest be detected and filled.

Dr. Newkirk mentions that in his paper, but I am glad to have the opportunity to emphasize it a little. What Dr. Newkirk says about the radical grinding of frail walls before filling I heartily agree with. Care in this line frequently prevents serious fracture.

*The President.*—Dr. Newkirk's paper is very interesting in the suggestions between, as well as in the lines themselves. The filling of small cavities so as to prevent them from becoming larger is a very desirable preventive measure. The examination of teeth I am sure all of you gentlemen have observed is frequently not thorough and careful enough to discover cavities that should have their progress arrested. The mechanical cleansing of the surfaces of the teeth, we all must agree, is the best of prophylactic measures against decay, and in the reference to chemical treatment of superficial decay you will probably observe that Dr. Newkirk means the use of nitrate of silver, as it is yet the only agent we can rely upon for arresting decay by chemical action. All of these measures are preventive, and they cannot be too strongly emphasized.

*Dr. Kimball.*—I had a suggestive case in my office to-day. A gentleman, a friend of mine, asked me if I would look at a Chinaman's teeth, a friend of his, and give him advice regarding them. Having some spare time to-day, I sent for him. His teeth were the best possible awful example I have ever seen of the result of neglect. The teeth were of good material and were strong. There was little or no decay, but the tartar had accumulated around them and they were covered with a brown serumal deposit. The teeth were loosened; some had already been lost. Pyorrhœa prevailed. The condition of his mouth was perfectly shocking.

The point in Dr. Newkirk's paper which I would wish to emphasize is that frequent and regular cleansing on the part of the dentist is a preventive, almost a total preventive, of pyorrhœa. That has been brought before us quite a number of times, but I think we may be pardoned for bringing it up again and emphasizing it. It has been my experience in a great many cases that we do not have pyorrhœa where the teeth are kept clean from childhood, so that the line of work outlined by Dr. Newkirk, the careful watching of the teeth and their cleansing by various means, such as the methods advocated by Dr. Smith and others, not only is a preventive of decay but of the much more serious disease, pyorrhœa, which causes not the loss of the individual parts of the teeth, but the loss of the tooth as a whole. I wish to emphasize with all the strength I have that one point. The cleansing of the teeth prevents pyorrhœa.

*Dr. F. Milton Smith.*—I have been considerably interested in reading the *résumé* and in the discussion of the paper. One or two points especially have impressed me. One was called attention to by our president,—the importance of finding minute cavities in children's teeth and the prevailing habit with many dentists of overlooking these small places. I understand that Dr. Davenport, who is almost always right, says Dr. Bogue finds all these little cavities and fills them for children. I am glad that Dr. Bogue stands by Dr. Davenport, even if he is wrong. I confess, with all due respect to these experienced men, that I do not always fill them, even if I do find them. I would like to ask Dr. Bogue if he would fill cavities in the teeth of a little boy eight years of age, whose upper incisors are not quite half-way through the gum and yet have cavities on the approximal surfaces. Would it be better to cut these cavities out and fill them, they not having gone through the enamel, or would it be better to smooth them off with disks and thoroughly polish the teeth?

Regarding the suggestion of finding cavities, I have never been so thoroughly impressed with its importance as in the past year, during which there has come into my hands a family of three girls, the youngest fifteen and the eldest twenty. The mother told me that they had regularly gone to a dentist, and that their teeth were supposed to have been put in thorough order three months before they came to me. I think in neither of these mouths have I found less than fifty cavities. It is simply amazing to me. Many appear to be tiny little black spots, but many times they turn out to be in size that of two or three pinheads. I am finishing with the youngest now, and I believe I have filled every approximate surface on the bicuspid teeth in the upper jaw, the fissures in all the bicuspids, all the fissures in the molars, and the approximate spaces in many of the molars. In one or two places exposed pulps were giving trouble. I have them now in a condition where I feel that, if they will take the best of care of their teeth and visit me every three months, it will be possible to save them.

*Dr. E. A. Bogue.*—I certainly intended no offence to Dr. Babcock, but if the president will allow I would like to read from Dr. Newkirk's paper.

"The first permanent molar next comes in for consideration. The longer I am in practice the more do I realize the importance of this abused member. I have seen within a week a case where by six months' neglect the pulp and nearly half the crown of a lower first molar were lost in the mouth of a boy eleven years old. Another where by the early extraction of the two lower first molars the third molars have erupted at an angle of about twenty-five degrees, the second molars have decayed badly next to them, and the whole occlusion of the mouth is unsatisfactory."

Dr. Davenport brought up the history of the young lady whose teeth erupted with seven or eight cavities in each. Very curious things occur in this world. It is a curious fact that cleft-palate patients seldom or never have any surplus money, and I have also noticed that people with whole lots of cavities in their teeth never have any energy. You may think this is funny, but children whose teeth are in process of formation and who are broken down physically, so that they are unable to assimilate properly, who may be undersized, and so lax that you cannot spur them up to do anything, almost invariably erupt teeth that are imperfect. I know such a girl who is twenty-three or twenty-four years of age. She is now married, and has spruced up a great deal and keeps her teeth much cleaner. I can excuse a great deal in her because I know it is difficult for her to get beyond this very deliberate way. Dr. Davenport, of Paris, made me feel very proud one day. He made a little speech at a dental dinner, and in that speech he said that he had been intimately associated with me for ten years, and in that time he had never seen a case of pyorrhœa among my regular patients, because I had insisted upon their keeping their teeth clean. Our late good friend Dr. Bronson gave me my first lesson in careful examination.

Now, to get down to Dr. Smith's question. He asked Dr. Davenport if he understood that I filled even the minutest points of *decay* in children's teeth? I promptly answer, "Yes." Then he asks this question about the incisor teeth with proximal cavities. I think it quite probable that in a case of this kind I should fill these cavities of decay with nitrate of silver until the teeth are sufficiently erupted. I have a few times removed superficial proximal decay by polishing. I have gone so far, as an object-lesson, to impress the importance of cleanliness upon my patients, as to pass a floss-silk between the teeth and then accidentally hold it in proximity to the nose. The result is usually very good. When I say I fill these cavities it does not mean that I would drill in where there are no cavities at all.

*Dr. R. H. M. Dawbarn.*—Allow me to apologize for breaking in on the order of the exercises. Dr. Allan courteously invited me to discuss his paper, and after reading it I would not for anything have been absent. I think he puts his finger on a condition which verges on an insult to the whole profession. I think we ought to act together. His paper, it seems to me, is a particularly broad and excellent one. While it discusses only the subject of the tariff, the attitude of our government towards our profession in other ways should be mentioned. This is simply one of indifference; and the attitude of the public is like that of the government. We have, in a way, ourselves to thank for it, since we by indifference render ourselves a negligible political factor. There is probably no public in the world that has so small a respect for the educated men of its medical profession as the American public. I had that brought directly home to me on the occasion of the illness of Rudyard Kipling. You all remember the fight he had for his life with pneumonia. At one time or another every portion of his lungs was, so I have been told, as solid as the liver. By the exercise of the most consummate medical skill his life was spared. Now, I think that in almost any other country in the world some word of praise would have been heard in the lay papers with regard to the ability in the handling of his case shown by his physicians. In not one paper in this city, where he was ill, was the matter alluded to, so far as I observed, and I looked for it persistently. On the contrary, one and all of the newspapers commented upon his courageous fight for his life—as if that alone could have saved him!

As regards the question of taxation, it is true that we are taxed tremendously, worse than in any other country in the world on everything in the way of surgical instruments. Skeletons are not taxed. I am sure I do not know why, unless the government does have a little hesitation in taxing dead men's bones. (Skeletons are almost invariably imported. They come from Paris and Vienna chiefly.) But to look at the subject in a broader sense, not alone in regard to the tariff, take the topic selected by Dr. Gerster, then president of the New York Surgical Society, in his annual address. He discussed the utter negligence of the American public in regard to its financial duties towards physicians. It is a fact that in New York City, as recently proved by legislative investigation, nearly one-half of the entire population are treated free of charge in the dispensaries and hospitals, and that estimate does not include those treated free in doctors' offices nor in the tenements. He called attention to the disgraceful fact that in American hospitals the visiting physicians and surgeons are not paid one penny; whereas abroad there is hardly a hospital where the reverse is not the case. In England, France, and Germany there is a decent compensation for the time of these physicians and surgeons; and in return the managers require regular attendance, which here they do not always get. I am glad to say that your branch of our profession is at last being recognized in hospital work, and now in at least two of our greatest hospitals (the New York City and the Kings County) dental surgeons are members of the staff; and if the time ever comes when physicians and surgeons are compensated, they will be compensated too. In London the greatest hospitals have visiting dentists. At the present time these dental surgeons in the New York hospitals occupy the same positions as the pathologist; they have all other privileges of the medical board, but they are not allowed to vote.

For the public lack of attention to its duty towards the medical profession we are ourselves partly to blame. Dr. Allan has stated that there are twenty-five thousand dentists in this country; and there are probably also one hundred and fifty thousand physicians. If all these, or even a large fraction of them, could work in harmony, think what a power we could wield politically, particularly in this matter of the tariff! It is especially so now, as the time has come, as President McKinley said in his last speech, when a tendency towards a diminution in the tariff duties seems to be in order. It has long been my hope to see, in matters affecting the common good of the medical profession, more uniformity of action. The present attitude seems to be that those who are the "outs" as to hospital appointments are pleased rather than the reverse that those who are the "ins" do not receive any salary. And upon the tariff physicians vote without thought as to the interests of their profession. That attitude we can overcome, I think, only in one way,—by every medical society constituting itself a political camp; not to discuss Republicanism or Democracy,—for I should deplore bringing up non-professional discussions,—but for the purpose of trying to induce its members to act together, harmoniously, and with force because of members, whenever matters affecting our common welfare as doctors are subject to public ballots. The tariff is surely one of these.

An invitation to the Institute to attend a meeting of the Second District Dental Society was read by the secretary.

Upon motion, the invitation was accepted.

Adjourned.

FRED. L. BOGUE, M.D., D.D.S.,
*Editor The New York Institute of Stomatology.*

The annual meeting of the Institute was held on Tuesday evening, December 3, 1901, at the office of Dr. S. E. Davenport, No. 51 West Forty-seventh Street, New York. The following officers were elected for the year 1902:

President, Dr. J. Morgan Howe; Vice-President, Dr. A. H. Brockway; Treasurer, Dr. J. Adams Bishop; Recording Secretary, Dr. F. Milton Smith; Corresponding Secretary, Dr. George A. Wilson; Curator, Dr. J. G. Palmer; Editor, Dr. Fred. L. Bogue.

*Executive Committee.*—Dr. S. E. Davenport, Chairman; Dr. C. O. Kimball, Dr. C. F. Allan.

